# Behavior change stages related to physical activity in adolescents from Santa Catarina: prevalence and associated factors

**DOI:** 10.1016/j.rppede.2016.03.017

**Published:** 2016

**Authors:** Jaqueline Aragoni da Silva, Kelly Samara da Silva, Adair da Silva Lopes, Markus Vinícius Nahas

**Affiliations:** Universidade Federal de Santa Catarina (UFSC), Florianópolis, SC, Brazil

**Keywords:** Physical activity, Exercise, Adolescent, Behavior, Theoretical models, Epidemiology

## Abstract

**Objective:**

Verify the prevalence and sociodemographic and economic factors associated with behavior change stages for habitual physical activity practice in adolescents from Santa Catarina.

**Methods:**

Secondary analysis of a study on the Behavior of Adolescents from Santa Catarina (CompAC 2). Cross-sectional school-based study of 6,529 high-school students (males, n=2,903) from the state of Santa Catarina public education system in 2011, aged 15 to 19 years. Multinomial logistic regression (crude and adjusted) was used to measure the association.

**Results:**

The highest and lowest prevalence rates were found in the maintenance (43.9%) and precontemplation stages (7.0%), respectively. The stages of action, preparation and contemplation showed similar results: 16.2%; 17.0% and 15.6%; respectively. Male adolescents show higher prevalence in the maintenance stage in relation to females and these show a higher prevalence in preparation, contemplation and precontemplation. All the assessed variables (gender, age, area of residence, employment status, family income, maternal education and school grade), with the exception of school shift, were associated with at least one of the stages.

**Conclusions:**

A large proportion of adolescents are in the pre-adoption stages and most of these have the intention to start regular physical activity. With the exception of school shift, the assessed variables were associated with stages in different ways, showing different profiles in relation to sociodemographic and economic characteristics in each subgroup.

## Resumo

**Objetivo:**

Verificar as prevalências e os fatores sociodemográficos e econômicos associados aos estágios de mudança de comportamento para prática habitual de atividade física em adolescentes catarinenses.

**Métodos:**

Análise secundária de estudo sobre o Comportamento do Adolescente Catarinense (CompAC 2). Estudo transversal de base escolar composto por 6.529 escolares (sexo masculino, n=2.903) do ensino médio da rede pública estadual de Santa Catarina em 2011, entre 15 a 19 anos. Para medida de associação, usou-se regressão logística multinomial (bruta e ajustada).

**Resultados:**

As prevalências mais elevadas e mais baixas foram verificadas nos estágios de manutenção (43,9%) e pré-contemplação (7%), respectivamente. Os estágios de ação, preparação e contemplação apresentaram resultados semelhantes entre si: 16,2%, 17% e 15,6%, respectivamente. Adolescentes do sexo masculino estão em maior prevalência no estágio de manutenção em relação aos do sexo feminino e essas apresentam maior prevalência em preparação, contemplação e pré-contemplação. Todas as variáveis investigadas (sexo, idade, área de moradia, situação ocupacional, renda familiar, escolaridade materna e série escolar), com exceção do turno de estudo, apresentaram associação com pelo menos um dos estágios.

**Conclusões:**

Grande parcela dos adolescentes encontra-se nos estágios de pré-adoção e a maioria desses tem a intenção de iniciar a prática regular de atividade física. Com exceção do turno de estudo, as variáveis investigadas estiveram associadas aos estágios de diferentes formas, apresentaram perfis distintos em relação às características sociodemográficas e econômicas em cada subgrupo.

## Introduction

There is a growing interest in the understanding of behavior related to physical activity (PA),^1^ due to the recognition of its importance in health maintenance.^2^ Given the fact that patterns of PA practice in adolescence tend to continue into adulthood,^3^ studies that investigate the behavior of young individuals is of great importance. Additionally, several theories have contributed to improve the understanding of PA-related behavior, given its complexity.^4^ The transtheoretical model is one of them, consisting of four constructs of which Stages of Behavior Change (SBC) are the most often researched.^5^

The SBC recommends that the individual follow stages, moving forward and/or backward within them to achieve a certain desired behavior, such as, for instance, regular PA practice.^6^ The model aims to elucidate motivational, behavioral and temporal aspects of the individual to change behavior.^7^ This model has advantages, such as the identification of both the individual's willingness, as well as the transition to PA practice.^8^ The effectiveness of interventions on PA based on this model has been demonstrated,^9^ as it is possible to classify in more details the individual's intent and differentiate those who are willing to change their behavior from those who are not.^6^ As for the stage, individuals in precontemplation do not plan to change their behavior, i.e., there is no intention to start PA practice. As for the contemplation stage, individuals think about the possibility of change, within a period of approximately six months. Those in the preparation stage have certain goals to start PA practice in the near future, set at 30 days. Individuals who are in the action stage have recently started the regular practice of PA. When in maintenance, individuals have practiced PA for more than six months, that is, they have achieved their behavior change.^7^

Studies show that each stage has its particular characteristics.^8^ Thus, in addition to knowing the SBC in which the adolescent is, we must also know what is associated with each stage, clarify external aspects (environment where the subject is) and what factors are characteristic at each level of PA-related behavior.^10^ Evidence suggests that sociodemographic indicators can play an important role in relation to SBC.^11^ In relation to gender and age, studies^12^^,^ 13 indicate that girls and older male adolescents are classified in the pre-contemplation, contemplation and preparation stages, whereas boys and younger adolescents are in the stages of action and maintenance.

It is noteworthy that, due to the size and cultural diversity of Brazil, different levels of PA-related behavior can be verified according to the individual's place of residence (region, state). Thus, studies with representative samples of adolescents allow us to extrapolate the results and help to define the profile of this population in different locations.

Therefore, the present study aimed to estimate prevalence and assess sociodemographic and economic factors associated with SBC for usual PA practice in adolescents from the state of Santa Catarina, Brazil. These questions may clarify existing gaps, assist in future interventions and allow actions directed at the characteristics of each subgroup.

## Method

Secondary analysis of the CompAC 2 project (Behavior of Adolescents from Santa Catarina), which portrayed the lifestyle and risk behavior of young individuals in Santa Catarina in 2011. This is a school-based epidemiological survey consisting of high-school students aged 15-19 years, attending public high schools in the state of Santa Catarina, Brazil. The project was submitted to the Institutional Review Board of Universidade Federal de Santa Catarina and obtained its approval (Process n. 1029/2010).

Based on the estimate of the adolescent population in Santa Catarina (205,572) in 2010,^14^ the sample was stratified according to six geographical regions, taking into account the distribution of Regional Education Secretariats. Lots were draw in two stages (size of schools and classes - school grade and shift). As several research phenomena were addressed, the following statistical parameters were considered: unknown prevalence (*p*=50%); 95% confidence interval and sample error of two percentage points. The sample design effect was also considered (*deff*=2), as well as a percentage of 25% for possible losses or refusals during data collection. The final sample size consisted of 6175 adolescents. The study had 6569 students as participants; however, there was a loss of 40 questionnaires (incomplete data). Only those who answered the questions related to SBC, or a total of 6507 adolescents, were part of this study.

The applied questionnaire was previously tested.^14^ Demographic (gender, age, residence area, employment status, school shift, grade) and economic (family income, maternal education) data were assessed. The following question was used to assess SBC: “A young individual is considered to be physically active when he/she accumulates at least 60min of moderate to vigorous PA on five or more days a week. Regarding your PA practice habits, you would say: (a) I have been physically active for more than six months (maintenance); (b) I have been physically active for less than six months (action); (c) I am not, but I intend to become physically active within the next 30 days (preparation); (d) I am not, but I intend to become physically active in the next six months (contemplation); (e) I am not and do not intend to become physically active in the next six months (pre-contemplation)”.

Descriptive statistics were used (absolute and relative frequencies) and 95% confidence intervals for the prevalence in the SBC. Multinomial logistic regression (crude and adjusted) was also used to analyze the associations between independent variables and outcome. The maintenance stage was treated as the reference category. In the adjusted analysis, a *p*<0.20 was considered for the permanence of the variables in the final model and the hierarchical model was used on three levels (distal level: gender and age; intermediate level: residence area, employment status, family income and maternal education; proximal level: school grade and shift).

## Results

Table 1 shows the sociodemographic and economic characteristics of the study sample. Of the 6507 adolescents, most were female (57.8%) aged between 15 and 16 years (58.8%) lived in urban areas (80.4%) and were employed (50.6%). There was a higher proportion of students with family income of three to five minimum wages (50.3%) and maternal educational level up to elementary school (54.4%). Regarding the school year, the majority of the adolescents were on 11th grade (36%), with most of the students attending the day shift (74.3%).

**Table 1:** Sociodemographic and economic characteristics of adolescents from public high schools in Santa Catarina (2011).

Variables	n	%
*Gender*		
Male	2903	42.2
Female	3626	57.8

*Age*		
15-16 years	3839	58.8
17-19 years	2690	41.2

*Area of residence*		
Rural	1537	19.6
Urban	4946	80.4

*Employment situation*		
Works	3656	50.6
Does not work	2870	49.5

*Family income*		
Up to three minimum wages	2155	31.3
Three to five minimum wages	3167	50.3
Six or more minimum wages	1131	18.4

*Maternal level of schooling*		
Elementary school	3612	54.4
High school	1879	33.1
College/university	684	12.5

*School grade*		
10th grade	2025	31.7
11th grade	2341	36.0
12th grade	2163	32.3

*School shift*		
Morning	3945	74.3
Evening	2584	25.7

The overall distribution of adolescents according to SCB indicated a higher prevalence in the maintenance stage (43.9%; 95%CI: 42-45.8). The stages of action (16.2%; 95%CI: 15.1-17.4), preparation (17%; 95%CI: 15.5-18.5) and contemplation (16%; 95%CI: 14.5-17.5) showed similar prevalence, whereas pre-contemplation (7%, 95%CI: 6.5-7.8) had a significantly lower prevalence than the other stages. When analyzed by gender, there was a higher prevalence in the maintenance stage in males compared to females. In the action stage, there were no statistically significant differences. On the other hand, there was a higher prevalence of girls in the stages of preparation and contemplation in relation to boys. Similarly, there was also a higher prevalence of girls than boys in the pre-contemplation stage.

Table 2 shows the distribution of adolescents by SBC according to sociodemographic factors. In the maintenance stage, a higher prevalence of adolescents was found for those who worked compared to those who did not work, as well as among adolescents whose family earned more than six minimum wages in relation to other income categories. As for the action stage, we found a higher prevalence among 10th grade students in relation to the 12th grade ones. In the preparation stage, there was a higher proportion of those with family income up to three minimum wages when compared to adolescents whose families earned more than six wages. In the contemplation stage, there was a higher prevalence of adolescents who did not work in relation to their peers. Moreover, 12th grade students showed a higher prevalence when compared to the other grades. Finally, in the pre-contemplation stage, adolescents who did not work showed a higher prevalence than those who did.

**Table 2:** Prevalence of adolescents at the behavior change stages according to sociodemographic and economic factors.

	n	Maintenance	Action	Preparation	Contemplation	Pre-Contemplation
		% (95%CI)	% (95%CI)	% (95%CI)	% (95%CI)	% (95%CI)
Variables		43.9 (42.0-45.8)	16.2 (15.1-17.4)	17.0 (15.5-18.5)	16.0 (14.5-17.5)	7.0 (6.3-8.0)
*Gender*						
Male	2.894	57.9 (55.5-60.3)	14.8 (13.3-16.5)	10.7 (9.3-12.2)	11.1 (9.6-12.9)	5.4 (4.3-6.8)
Female	3.613	33.6 (31.0-36.4)	17.2 (15.6-19.0)	21.6 (19.6-23.7)	19.5 (17.5-21.6)	8.1 (7.2-9.1)

*Age*						
15-16 years	3.823	43.7 (42.1-45.2)	18.0 (16.8-19.2)	17.1 (15.9-18.3)	14.2 (13.1-15.3)	7.1 (6.3-7.9)
17-19 years	2.684	47.6 (45.7-49.5)	13.7 (12.4-15.0)	15.2 (13.9-16.6)	16.2 (14.8-17.6)	7.2 (6.2-8.2)

*Area of residence*						
Rural	1.532	45.8 (41.1-50.6)	14.0 (11.6-16.7)	17.6 (15.6-19.9)	15.3 (12.5-18.6)	7.3 (5.8-9.0)
Urban	4.930	43.4 (41.5-45.4)	16.7 (15.4-18.1)	16.9 (15.2-18.7)	16.1 (14.5-17.9)	6.9 (6.1-7.8)

*Employment situation*						
Works	3.640	50.2 (48.0-52.3)	16.2 (14.9-17.6)	15.0 (13.5-16.6)	13.4 (12.0-15.0)	5.2 (4.5-6.1)
Does not work	2.865	37.5 (34.8-40.4)	16.2 (14.6-18.0)	19.0 (16.6-21.7)	18.5 (16.5-20.8)	8.7 (7.6-9.9)

*Family income* ^a^						
Up to two	2.148	40.9 (37.6-44.3)	16.9 (14.7-19.3)	19.3 (17.5-21.4)	14.6 (12.8-16.6)	8.3 (6.9-10.1)
Three to Five	3.160	43.2 (40.2-46.3)	16.4 (14.8-18.2)	17.1 (14.7-19.7)	16.9 (14.9-19.0)	6.4 (5.7-7.3)
Six or more	1.128	50.6 (47.1-54.1)	14.7 (12.6-17.0)	12.6 (10.3-15.2)	15.9 (13.7-18.5)	6.3 (4.6-8.4)

*Maternal level of schooling*						
Elementary school	3.600	43.2 (40.8-45.6)	15.9 (14.6-17.2)	18.0 (16.3-19.8)	16.1 (14.0-18.3)	6.9 (5.9-8.1)
High school	1.878	44.1 (41.0-47.2)	16.7 (15.0-18.6)	15.7 (13.5-18.1)	17.0 (14.9-19.4)	6.6 (5.5-7.8)
College/university	683	48.3 (44.2-52.3)	15.0 (12.3-18.3)	16.5 (12.4-21.8)	13.2 (10.5-16.4)	7.0 (5.5-8.9)

*School grade*						
10th	2.014	44.7 (41.6-47.8)	18.3 (16.2-20.5)	16.9 (15.4-18.6)	11.9 (9.6-14.7)	8.2 (6.7-10.0)
11th	2.331	43.8 (41.0-46.6)	17.2 (15.1-19.5)	17.3 (15.0-19.8)	15.2 (13.0-17.8)	6.5 (5.5-7.7)
12th	2.162	43,2 (40.2-46.3)	13.1 (11.4-15.1)	16.7 (14.6-19.1)	20.7 (18.7-22.9)	6.3 (5.1-7.7)

*School shift*						
Morning	3934	42.9 (40.6-45.3)	16.4 (15.1-17.7)	17.2 (15.4-19.2)	16.5 (14.8-18.3)	7.1 (6.2-8.2)
Evening	2573	46.7 (44.2-49.3)	15.8 (13.7-18.1)	16.4 (14.6-18.4)	14.5 (12.6-16.6)	6.6 (5.4-8.1)

CI, confidence interval.

a Minimum wages.

The results for the crude and adjusted multiple analysis (reference category: Maintenance) showed similarities and can be seen in Tables 3 and 4. After adjusting the data, it was found that there was a greater chance for adolescent girls, younger individuals and those residing in urban areas to be in the action stage. Associations were found in the preparation stage between female adolescents, adolescents who did not work and those belonging to the lower income strata (in comparison to the higher). As for contemplation, there was a greater chance of being at this stage for girls, adolescents who did not work, those with higher levels of income and those with maternal education up to elementary school (when compared to those with higher level of education) and those attending 12th grade (in relation to 10th grade). Finally, a higher chance of being in the pre-contemplation stage was found in girls and adolescents who did not work.

**Table 3:** Crude odds ratio for behavior change stages according to demographic and economic factors.

Variables	Action	Preparation	Contemplation	Pre-contemplation
	Crude OR (95%CI)	Crude OR (95%CI)	Crude OR (95%CI)	Crude OR (95%CI)
*Gender*				
Male	1	1	1	1
Female	2.00 (1.64-2.43)	3.47 (2.85-4.23)	3.01 (2.41-3.76)	2.56 (1.93-3.40)

*Age*				
16-17 years	1	1	1	1
18-19 years	0.73 (0.61-0.87)	0.84 (0.70-1.01)	1.08 (0.09-1.29)	0.87 (0.68-1.12)

*Area of residence*				
Rural	1	1	1	1
Urban	1.26 (0.96-1.66)	1.01 (0.81-1.27)	1.11 (0.83-1.49)	1.00 (0.76-1.34)

*Employment situation*				
Works	1	1	1	1
Does not work	1.34 (1.16-1.55)	1.70 (1.35-2.14)	1.84 (1.55-2.19)	2.22 (1.79-2.77)

*Family income* ^a^				
Up to two	1	1	1	1
Three to five	0.92 (0.72-1.17)	0.83 (0.64-1.08)	1.10 (0.87-1.38)	0.73 (0.56-0.96)
Six or more	0.70 (0.52-0.94)	0.52 (0.41-0.68)	0.88 (0.68-1.14)	0.61 (0.42-0.89)

*Maternal level of schooling*				
Elementary school	1	1	1	1
High school	1.03 (0.87-1.23)	0.85 (0.69-1.05)	1.04 (0.82-1.32)	0.93 (0.72-1.19)
College/university	0.85 (0.67-1.07)	0.82 (0.59-1.16)	0.73 (0.54-1.00)	0.91 (0.69-1.20)

*School grade*				
10th	1	1	1	1
11th	0.96 (0.76-1.22)	1.04 (0.86-1.26)	1.31 (0.94-1.81)	0.81 (0.59-1.11)
12th	0.74 (0.59-0.93)	1.02 (0.84-1.24)	1.81 (1.42-2.29)	0,.79 (0.59-1.06)

*School shift*				
Morning	1	1	1	1
Evening	0.88 (0.72-1.08)	0.88 (0.71-1.07)	0.81 (0.67-0.98)	0.85 (0.65-1.12)

CI, confidence interval; OR, Odds ratio.

a Minimum wages.

**Table 4:** Adjusted odds ratio for behavior change stages according to demographic and economic factors.

Variables	Action	Preparation	Contemplation	Pre-contemplation
	Adjusted OR (95%CI)	Adjusted OR (95%CI)	Adjusted OR (95%CI)	Adjusted OR (95%CI)
*Gender*				
Male	1	1	1	1
Female	1.98 (1.63-2.41)	3.46 (2.85-4.20)	3.02 (2.42-3.77)	2.55 (1.92-3.40)

*Age*				
16-17 years	1	1	1	1
18-19 years	0.75 (0.63-0.90)	0.88 (0.73-1.05)	1.21 (0.94-1.34)	0.90 (0.70-1.15)

*Area of residence*				
Rural	1	1	1	1
Urban	1.33 (1.01-1.76)	1.07 (0.83-1.38)	1.10 (0.81-1.50)	1.02 (0.76-1.40)

*Employment situation*				
Works	1	1	1	1
Does not work	1.16 (1.00-1.34)	1.41 (1.11-1.79)	1.71 (1.45-2.03)	1.89 (1.46-2.44)

*Family income* ^a^				
Up to two	1	1	1	1
Three to five	1.05 (0.84-1.31)	1.02 (0.78-1.33)	1.43 (1.18-1.74)	0.82 (0.61-1.11)
Six or more	0.84 (0.62. 1.12)	0.77 (0.60-0.99)	1.44 (1.10-1.89)	0.78 (0.54-1.21)

*Maternal level of schooling*				
Elementary school	1	1	1	1
High school	1.02 (0.86-1.23)	0.88 (0.70-1.11)	1.03 (0.81-1.32)	0.97 (0.76-1.25)
College/university	0.83 (0.66-1.04)	0.89 (0.64-1.24)	0.67 (0.48-0.94)	0.94 (0.68-1.29)

*School grade*				
10th	1	1	1	1
11th	0.97 (0.75-1.24)	1.04 (0.83-1.29)	1.35 (0.93-1.96)	0.86 (0.63-1.17)
12th	0.79 (0.61-1.03)	1.07 (0.80-1.43)	2.01 (1.47-2.75)	0.75 (0.54-1.04)

*School shift*				
Morning	-	-	-	-
Evening	-	-	-	-

CI, confidence interval; OR, Odds ratio.

a Minimum wages.

## Discussion

The findings indicate a higher prevalence of adolescents in the maintenance stage and a lower prevalence in the pre-contemplation stage, which is in accordance with results of studies carried out in two areas of Brazil.^15^ On the other hand, prevalence rates found in studies from other countries show divergent results. De Bourdeauhiuj^12^ found that of 5931 assessed adolescents, most were in the maintenance stage, but the lowest proportion was in the action stage. Kim^16^ investigated Korean adolescents and found that the stages with the highest and lowest prevalence were, respectively, action and contemplation. Disparities in results may be due to differences related to ethnic groups, which exert influence on the SBC.^17^ Furthermore, we point out the relevance of the measuring tools and how methodological differences can impact the results (questions with Likert scale format and organization chart, among others).^9^ To standardize what will be considered in relation to PA (PA domain, time, frequency, etc.) to classify in each stage is very important to allow comparability of data from different studies.^9^

Regarding the prevalence according to gender, the results found here are similar to other studies, in which boys are the mostly in the maintenance stage and girls in the preparation, contemplation and pre-contemplation stages.^16^ As for the action stage, studies show a higher prevalence in both the female^12^ and the male gender.^1^^,^ 13 ^,^ 16 In this study, although a higher prevalence was verified in girls, the results were not statistically significant.

Regarding factors that may explain the classification of the adolescents in SBC, it was found that gender is one of them. The chance of regularly practicing PA is higher for males. In fact, the literature is consistent in showing that boys are more active than girls.^18^ However, it is noteworthy that girls are approximately three times more likely to have the intent of changing behavior when compared to boys. According to Spence et al.,^19^ female adolescents have lower self-efficacy when compared to males. According to Prochaska,^20^ this factor significantly contributes to progression in SBC, which may explain the fact that while female adolescents have the intention to, psychosocial issues prevent them from changing their behavior. Thus, interventions that take into account adolescent gender differences may be more effective, with actions directed to the peculiarities of girls and boys.

Regarding age, according to the literature, older adolescents are at higher proportion in the pre-adoption stages, while younger ones are at the post-adoption stages.^1^^,^ 12 There are also some studies that have shown no association between these variables.^15^ In the present study, being an older adolescent (17-19 years) is a protective factor for the action stage, which indicates there is lower chance of recent incorporation of an active behavior (last six months) in this age group in relation to the younger one (15-16 years). These findings are consistent with the literature, which indicates that the end of adolescence is characterized by a decrease in PA levels.^21^^,^ 22 According to Ortega,^22^ this stage of life is characterized by both physiological and psychological changes. Moreover, the transition to adult life requires, for many, to leave their homes due to issues related to college/university and/or work, which leads to major changes in lifestyle.

As for the residence area, students from urban areas, when compared to those from rural areas, are more likely to be in the action stage than in the maintenance stage. These findings indicate that in urban areas there is a recent adoption of PA-related behavior; however the chance for the adolescent to maintain an active behavior is lower. Oliveira^13^ found no statistically significant differences between urban and rural areas when classifying adolescents according to SBC. However, it was observed that living in the coastal region was associated with post-adoption stages in relation to living in the countryside of the state of Pernambuco, Brazil. It can be observed, then, that the SBC classification may vary according to the place of residence, as even though the SBC originate from a primarily psychological theoretical model, the influence of the physical environment is acknowledged.^23^

Regarding the employment status, an association was observed between not working and the pre-adoption stages, i.e., without regular practice of PA. Similarly to these findings, Oliveira^13^ found that both girls and boys who work are more likely to be in the stages of action and maintenance. Studies that investigate the topic point out that the type of work performed by the adolescents most often involves physical exertion,^24^ which may favor their being in the stages of action or maintenance. Considering that adolescents who do not work have more free time, providing programs that encourage the practice of PA can be a strategy to help this population segment to adopt a more active behavior, as long as the interventions are specific for each stage.^25^

The literature shows that low income is associated with the pre-adoption stages.^1^ In this study, higher income is both a risk and protective factor for the maintenance stage, depending on the assessed subgroup. As for divergences in that study, Garber,^25^ when studying SBC correlates, concluded that the complexity related to the individual's intention to practice PA indicates the presence of potential mediators and/or confounding variables that differ, depending on the individual characteristics. Psychosocial issues, for instance, are different stages at which adolescents in contemplation perceive fewer benefits and have more PA-related barriers in relation to the preparation stage.^12^

Adolescents whose mothers have college/university education were more likely to be in the maintenance stage in relation to contemplation, when compared to those whose mothers have up to elementary school education. There are no studies in literature of which results found significant differences among adolescents.^13^ Adults with higher educational level are more likely to be active^26^ and their behavior and attitude have a strong influence on their children's habits.^27^ Based on this evidence, we emphasize the importance of including questions related to parental behavior when investigating PA-related behavior in adolescents, as they can lead to further explanations of this phenomenon.^28^

Regarding the school year, it was observed that attending the last year of high school is a risk factor for the contemplation stage. Of the studies that investigated the association between school year and SBC,^1^^,^ 16 only one showed there is a tendency toward a reduction in the levels of PA practice with the advance in school years.^1^ One of the reasons that can justify this reduction is the higher study load, due to the College/University admission tests.^29^ The same author, when studying barriers to PA practice, found that the barrier related to the studies was the one most often cited among students on the 11th and 12th grades.

To investigate SBC in adolescents makes it possible to develop more effective strategies, as targeted actions can be created for groups with different profiles. The model itself offers explicit suggestions on how to assist subjects who intend to change their behavior, according to the stage where they are.^30^ Therefore, this study has brought relevant contributions about the intentions and behavior of adolescents from Santa Catarina. Moreover, the questionnaire applied in the study was validated for use in this population. Furthermore, the sample size and its representative character allow the comparability of data with other studies of epidemiological scale and school-based ones involving adolescent students from public schools.

However, despite the relevant information found herein for the study area, it is important that the results be interpreted with caution due to some limitations inherent to the research. First, the cross-sectional design does not allow the inference of a causal association between some of the assessed variables. Another question is the fact that the measures are subjective ones, implying the presence of recall bias, as well as the phenomenon known as social desirability, which means that the individual does not always respond in fact about their actual behavior. Finally, this information does not reflect the reality of adolescents enrolled in private schools, as well as adolescents in this age group who do not attend school.

In conclusion, it can be said that many of the adolescents from the public school system in Santa Catarina are in the stages of pre-adoption of regular PA practice. The stages of precontemplation and maintenance showed, respectively, the lowest and highest prevalence of adolescents. There was a higher prevalence of boys in the maintenance stage and of girls in the pre-adoption stages, especially in stages characterized by intention to change. Moreover, we conclude that, in addition to gender, socio-demographic and economic factors are associated with SBC. The results found herein collaborate to a better understanding of the intent of adolescents from Santa Catarina regarding PA practice.
